# A biomechanical comparison of two screw fixation methods in a Letenneur type I Hoffa fracture

**DOI:** 10.1186/s12891-020-03527-4

**Published:** 2020-07-28

**Authors:** Shu-Hsin Yao, Wei-Ren Su, Kai-Lan Hsu, Yueh Chen, Chih-Kai Hong, Fa-Chuan Kuan

**Affiliations:** 1 Department of Orthopaedic Surgery, Ditmanson Medical Foundation Chia - Yi Christian Hospital, Chiayi, Taiwan; 2 Department of Orthopaedic Surgery, National Cheng Kung University Hospital, College of Medicine, National Cheng Kung University, Tainan, Taiwan; 3 Skeleton Materials and Bio-compatibility Core Lab, Research Center of Clinical Medicine, National Cheng Kung University Hospital, College of Medicine, National Cheng Kung University, Tainan, Taiwan; 4 Department of Biomedical Engineering, National Cheng Kung University, Tainan, Taiwan; 5 Department of Orthopaedic Surgery, Sin Lau Hospital, Tainan, Taiwan

**Keywords:** Distal femur fracture, Hoffa fracture, Internal fixation on, Biomechanics

## Abstract

**Background:**

The treatment of Hoffa fractures is challenging, for which the ideal fixation and approach are still controversial. Osteosynthesis with plate or screws fixation in different trajectories have been described in previous literature. The purpose of this study was to compare the biomechanical strength and stability of two types of screw trajectories used to stabilize displaced coronal fractures of the lateral femoral condyle.

**Methods:**

Sixteen synthetic femurs (Sawbones Pacific Research Laboratories, Vashon, WA) were divided into two groups. A vertical osteotomy was performed to mimic a Letenneur type I Hoffa fracture. Group A (*n* = 8) was fixed with screw in anteroposterior direction (A-P) screws. Group B (*n* = 8) was fixed with crossed screws. Both groups were tested with a nondestructive axial compression aligned with the femur axis. After that, 10,000 cyclic loading tests were applied to the specimen with a force ranging between 200 to 600 N, and the interfragmental displacement was recorded, respectively, after 10, 100, 1000 and 10,000 cycles. Finally, a destructive axial compression test was applied until catastrophic failure.

**Results:**

There were no statistical between-group differences in regard to the average axial stiffness, interfragmental displacement, and ultimate failure load. The average axial stiffness of the A-P screw was comparable to that of the crossed screw (361 ± 113 N/mm vs. 379 ± 65 N/mm, *p* = 0.753). All specimens completed the entire cyclic loading test without catastrophic failure, and the interfragmental displacement after loading for 10,000 cycles was 1.36 ± 0.40 mm for the A-P screw and 1.29 ± 0.61 mm for the crossed screw, there were no statistical differences between the groups (*p* = 0.823). The average ultimate failure loads for the A-P and crossed screws were 1214 ± 127 N and 1109 ± 156 N, respectively (*p* = 0.172).

**Conclusions:**

Based on our in vitro study, the crossed screws can provide comparable mechanical performance as traditional A-P screws in Hoffa fracture fixation. Considering the screws trajectories are commonly determined by the choice of surgical approach, the current study provides support from a biomechanical perspective for the application of crossed screws in direct lateral approach.

## Background

The Hoffa fracture, which was described by Hoffa first in 1904 [1], represents a fracture pattern that extends coronally in the distal femoral condyle. This type of intraarticular fracture only accounts for 8.7% ~ 13% of fractures occurring in the distal femur, and the incidence rate is higher in the lateral as compared to the medial condyle [2–6]. To prevent misdiagnosis, computerized tomography (CT) has been recommended to those sustaining a high-energy distal femoral fracture [7]. As a result of large shearing force, the Hoffa fracture is susceptible to displacement while bearing weight [8]. Nonoperative treatment is not recommended, given that the outcome is unsatisfactory [9]. The aim of surgical treatment is to establish anatomic reduction and rigid fixation [10–13].

The ideal fixation and approach to Hoffa fractures are still subject to debate [14–17]. Plate fixation has been shown to have better stability as compared to isolated screw fixation; however, some unavoidable risks, including extensive dissection and difficulty in plate contouring, have also been reported [8, 18]. In contrast, screw fixation offers an alternative option with minimal soft tissue dissection. Different screw trajectories, including A-P screws (anteroposterior direction), P-A screws (posteroanterior direction), or crossed screws have been described in previous studies, and all have their proponents [8, 11, 19–21]. The surgical approach also determines the screw trajectory. Given that the Hoffa fracture is diagnosed in association with 38.1% of supracondylar-intercondylar femoral fractures [4], a direct lateral approach (DLA) or parapatellar approach (PPA) is typically chosen to treat the associated fractures simultaneously [22, 23]. A-P screws and crossed screws can be smoothly utilized through the use of these approaches. Although P-A screws were shown to have better fixation strength than A-P screws in two biomechanical studies [8, 11], an additional posterior approach was necessary. Therefore, A-P screws and crossed screws are still widely used in combined Hoffa and distal femoral fractures. To the best of our knowledge, there are no studies that have directly compared these two types of screw trajectories in terms of biomechanics.

The purpose of this study is to compare the biomechanical characteristics of the A-P screws with the crossed screws in a Hoffa fracture. The hypothesis is that in the load-to-failure and cyclic loading test, the crossed screws will provide comparable mechanical properties to those of A-P screws.

## Methods

### Specimen preparation

Sixteen medium, left side, 4th generation synthetic composite femurs (model 3403, Sawbones, Vashon, WA) were utilized in the study. Each specimen was cut into a 18 cm distal segment and divided into two groups of 8 each. Then, the anatomic axis of the femurs were well-aligned in a 6 cm cylinder tube. Afterwards, the specimen was potted in a cylinder tube using anchoring cement (PMMA).

Each specimen was predrilled prior to the osteotomy to facilitate the subsequent anatomic reduction. Furthermore, a specific prefabricated drilling jig was utilized to ensure the consistency of guide pin placement. Initially, two 3.2 mm threaded pin were advanced under fluoroscopic guidance. After that, the screw length was determined with a direct measuring gauge. Finally, a 4.9 mm cannulated drill and a 6.5 mm tap were used to prepare the two screw holes.

In order to mimic the Letenneur type I Hoffa fracture, an oscillating saw was utilized to perform the lateral condylar osteotomy, which was inferiorly extend from the extraarticular condyle-shaft junction to the articular surface. The fracture line was parallel to the axis of posterior femoral cortex. Then, the fracture was reduced anatomically and secured with one of the two screw trajectories described below. All of the surgical procedures were performed by one surgeon under fluoroscopic guidance to ensure accurate implant length and position.

#### Group A

**Fig. 1:**
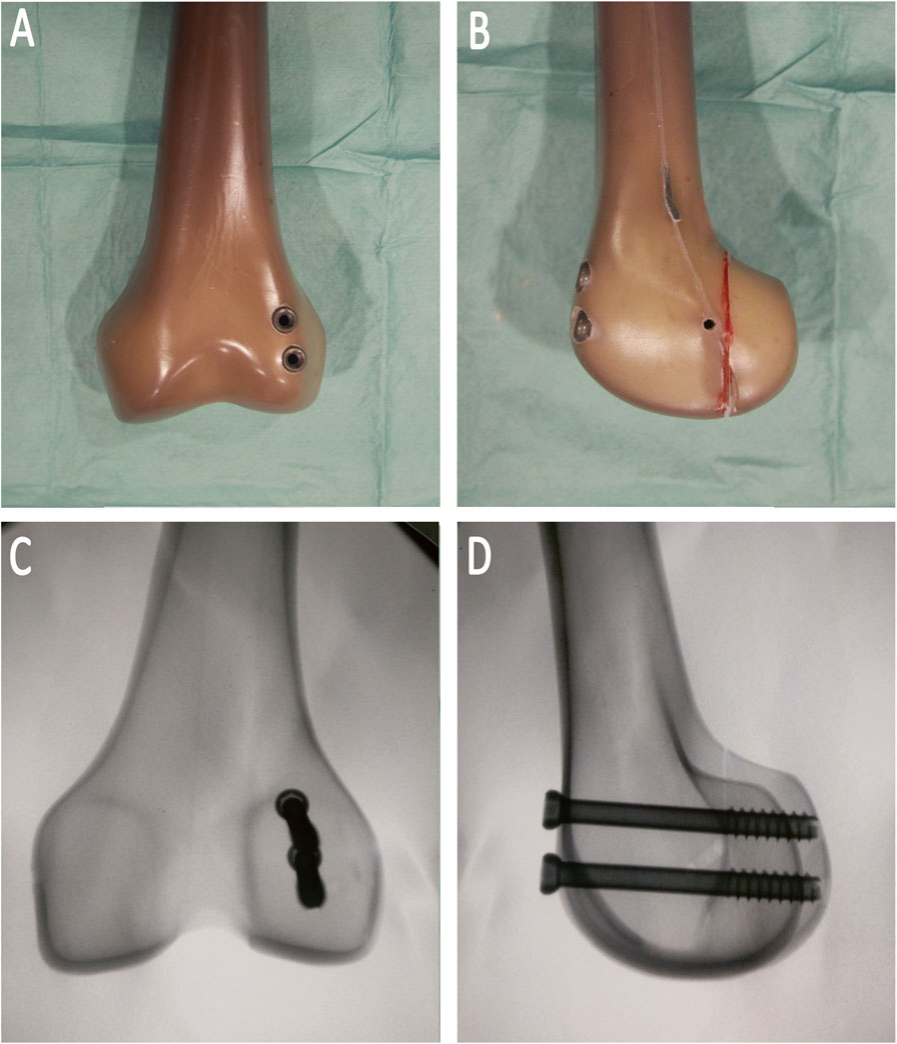
The specimens were repaired using A-P direction screws. **a** The dorsal view of the construct; **b** the lateral view; **c** The anteroposterior radiograph view (**d**) The lateral radiograph view

Two parallel 6.5-mm partially threaded cannulated screws (Stryker, Kalamazoo, MI) were placed in the A-P fashion. The screws were inserted from the nonarticular portion of the femoral trochlear and were directed posteriorly across to the fracture line perpendicularly. Then, 60 mm screws were chosen after measurement with a depth gauge. (Fig. 1).

#### Group B

**Fig. 2:**
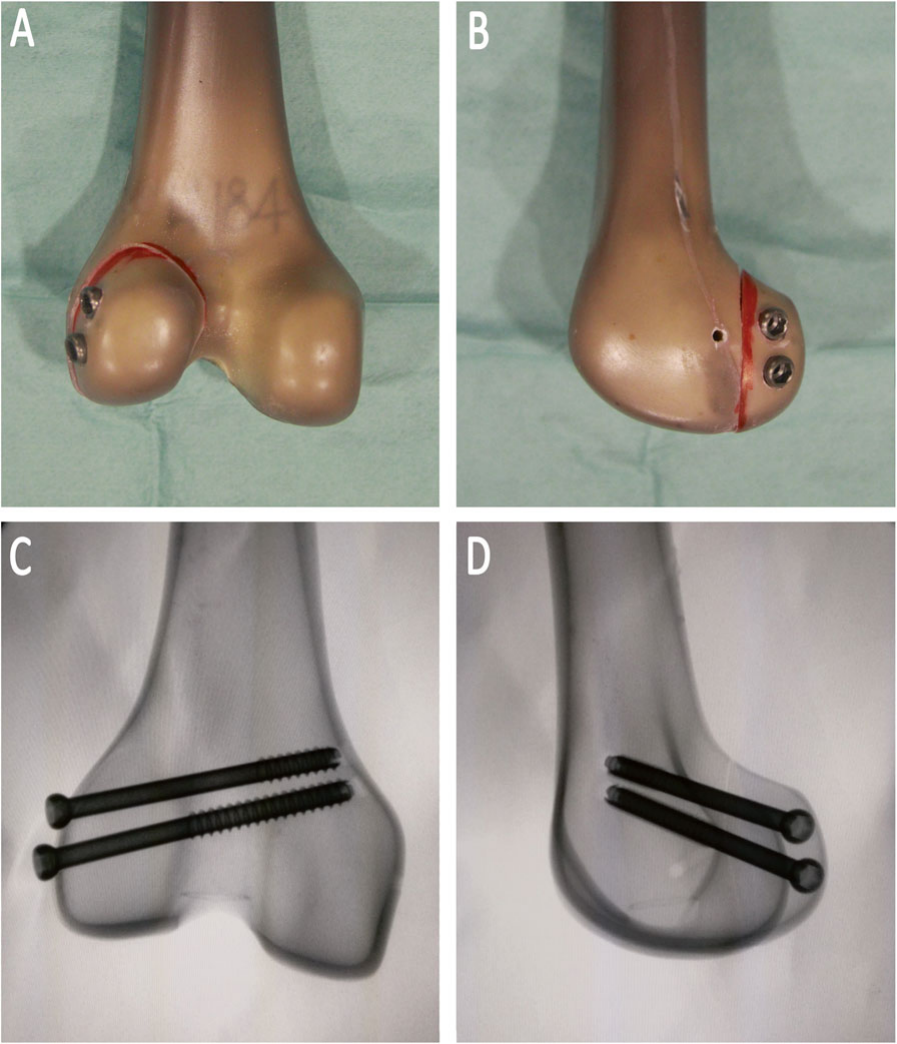
The specimens were repaired using crossed screws. **a** The dorsal view of the construct; **b** the lateral view; **c** The posterioanterior radiograph view; **d** The lateral radiograph view

Two parallel 6.5-mm partially threaded cannulated screws were placed in the crossed fashion. The screws were inserted from the nonarticular lateral aspect of the condylar fragment, aiming at an inclination of 30° proximally and 45° anteriorly. Through this trajectory, screw penetration to the patella-femoral joint could be prevent. Then, 70 mm screws were chosen after measurement with a depth gauge. (Fig. 2).

### Biomechanical protocol

**Fig. 3:**
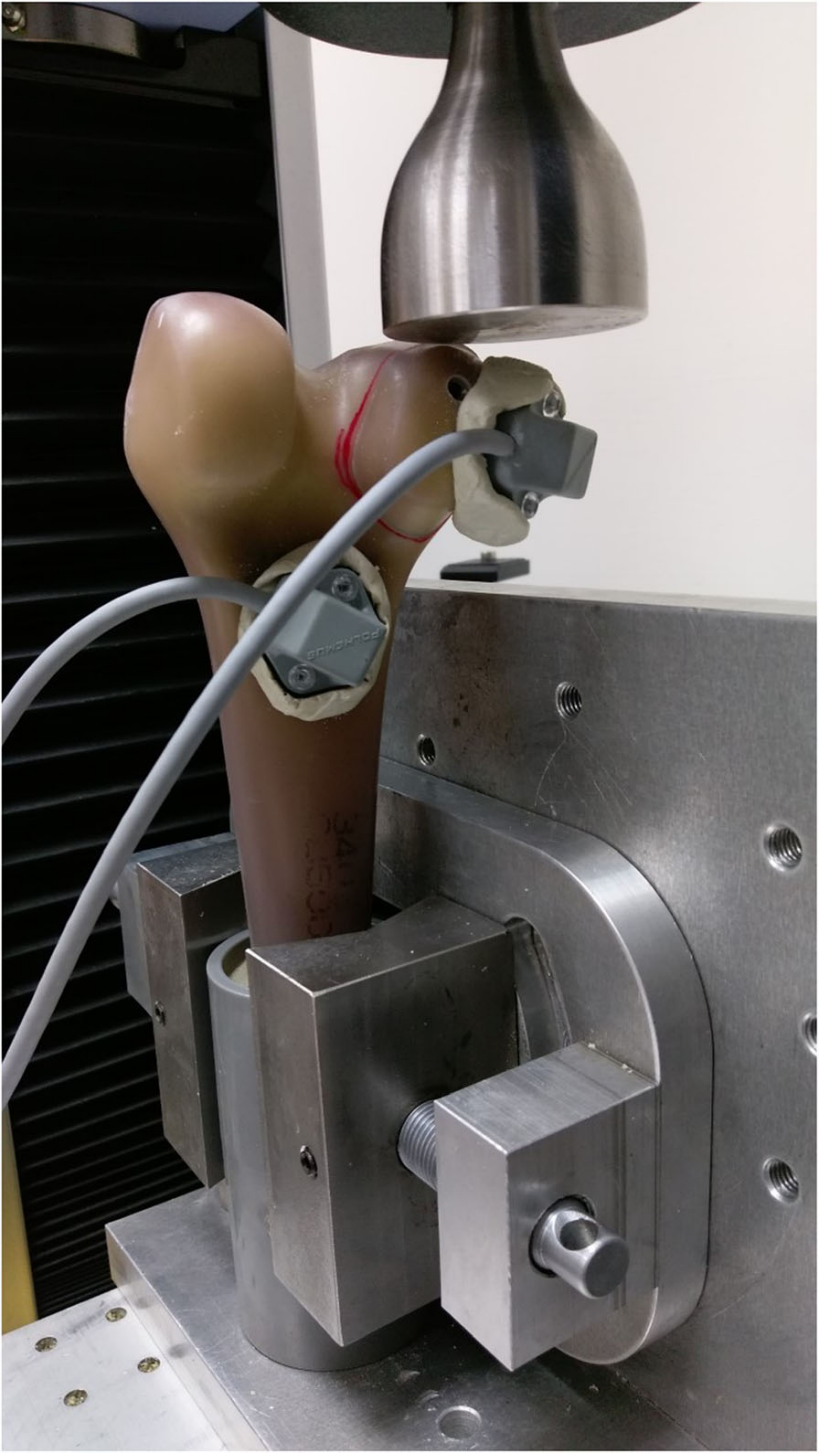
The biomechanical setup with a composite distal femur mounted on a metal clamp. The displacement sensors were secured with plastic screws and anchoring cement

A materials-testing machine (AG-X; Shimadzu Corp., Tokyo, Japan) was utilized for the biomechanical tests.. The specimen potted in a cylinder tube was secured distally into a fabricated adjustable metal clamp (Fig. 3). Afterwards, a flat stainless steel plate connected to a 5000 N load cell was centered on the Hoffa fracture to apply an axial compression force. Initially, a 100 N preload was applied to the specimens at a speed of 2 mm/min. Then, the load was increased to 300 N at a speed of 10 mm/min. The slope in the linearly elastic region of the load-displacement curve was recorded and calculated as the axial stiffness. Second, a 10,000 cycle repeated cyclic loading test was applied to the specimen with a force ranging between 200 and 600 N (valley/peak) at a frequency of 1 Hz, and the interfragmental displacement was recorded, respectively, after 10, 100, 1000, and 10,000 cycles. Finally, destructive axial compression was loaded at a speed of 10 mm/min on each specimen until catastrophic failure occurred, which might result from screw dislodging, fragment cracking or cortex disruption. The loading protocol mainly followed a previous study [11]. The maximum load detected by the testing machine during the load-to-failure test was defined as failure load. In addition, all failure modes were recorded for the purpose of the analysis. There were two sensors firmly fixed by cement onto the fragment and intact distal femur respectively. All the interfragmental displacement during the static and cyclic loading was measured with a magnetic tracking system (Polhemus, Colchester, VT, USA), by which the real-time data was detected at a resolution of 0.03 mm.

### Statistical analysis

Following the pilot study, in which three specimens in each group was conducted, an *priori* power analysis was performed using G*Power [24]. The effect sizes were calculated from the preliminary results and were 2.17 for the failure load and 1.18 for the stiffness. To provide a study power of 0.9 and α value of 0.05, the projected sample size needed with this effect size is approximately 8 for each group. The final data from the two groups was conducted using SPSS software (SPSS Version 17; SPSS Inc., Chicago, IL, USA). To determine the differences in axial stiffness and destructive failure load between the two types of screw trajectories, considering the small sample size, the Mann-Whitney U test was performed for the distribution-free equivalent statistic on the data analysis. As regards the cyclic displacement, considering that repeated measurements of cyclic displacement were made four times on each specimen, a 4-step General Linear Model Repeated Measures Test was performed. A confidence interval of 95% was used for all the parameters to determine significance.

## Results

The raw biomechanical data for axial stiffness, the interfragmental displacement at each specific cycle, and the ultimate failure load are listed in Table 1.

**Table 1:** Average axial stiffness, post cyclic load displacement, and ultimate failure load in the different groups

Groups	Stiffness (N/mm)	Displacement (mm)				Failure load (N)
		10 cycles	100 cycles	1000 cycles	10,000 cycles	
A-P	361 (113)	0.69 (0.19)	0.88 (0.23)	1.12 (0.25)	1.36 (0.40)	1214 (127)
Crossed	379 (65)	0.90 (0.48)	1.04 (0.55)	1.14 (0.62)	1.29 (0.61)	1109 (156)
*p*-value	0.753	0.265	0.454	0.938	0.823	0.172

Average values are given along with one standard deviation in parentheses

### Axial stiffness

The slope in the linearly elastic region was referred as axial stiffness, which was 361 ± 113 N/mm in the group A and 379 ± 65 N/mm in the group B. There were no significant differences between the groups (*p* = 0.753).

### Interfragmental displacement

The interfragmental fracture displacement after each specific cyclic loading was collected, where it was found that the value increased steadily during the cyclic test. In the group A, the interfragmentary fracture displacement after 10, 100, 1000, and 10,000 cycles was 0.69 ± 0.19 mm, 0.88 ± 0.23 mm, 1.12 ± 0.25 mm, 1.36 ± 0.40 mm, respectively. In the group B, the interfragmentary fracture displacement after 10, 100, 1000, and 10,000 cycles was 0.90 ± 0.48 mm, 1.04 ± 0.55 mm, 1.14 ± 0.62 mm, 1.29 ± 0.61 mm, respectively. There were no statistically significant differences between the two fixation constructs after each cycle.

### Load to failure

**Fig. 4:**
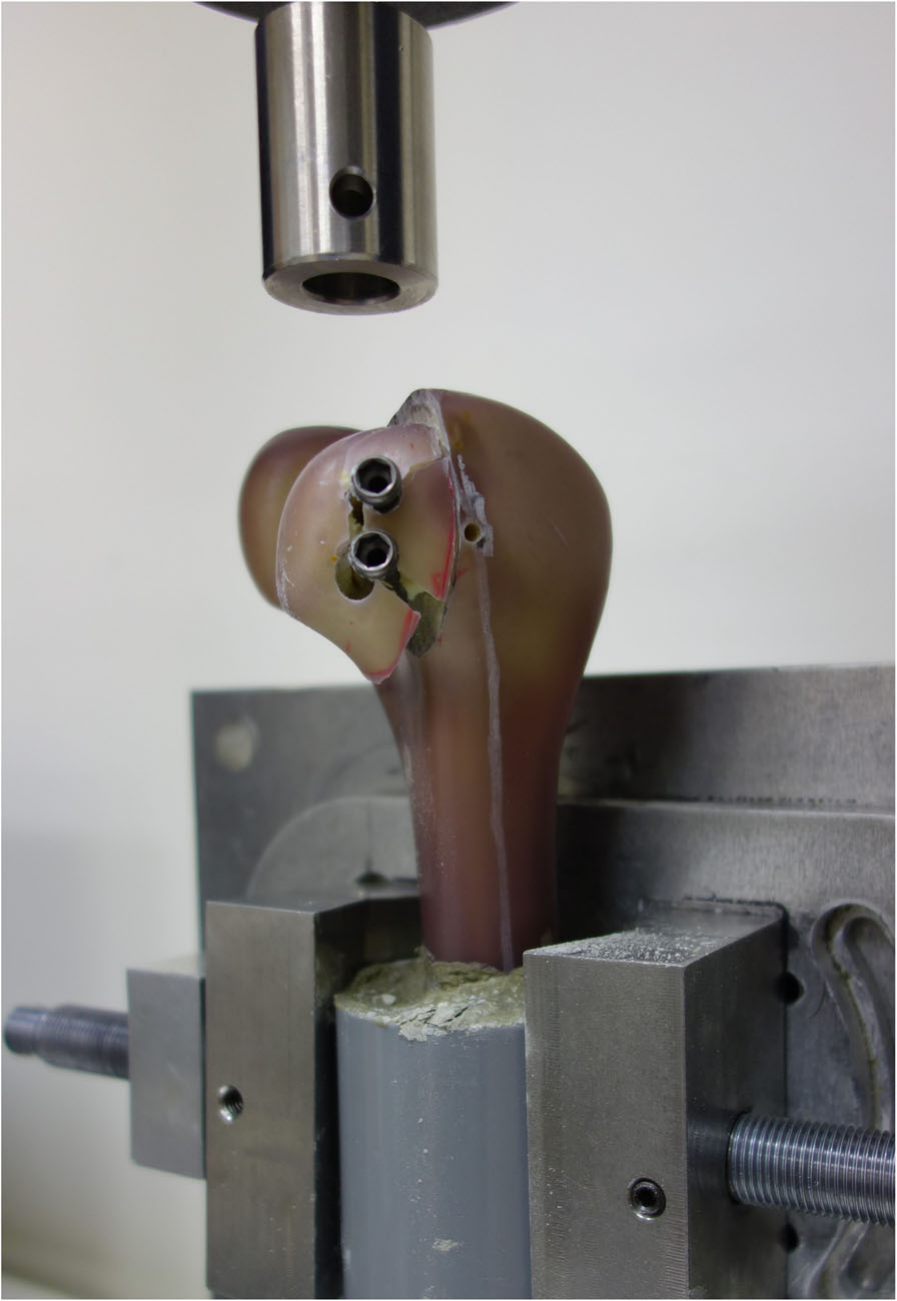
The Hoffa fragment was displaced distally from its original position. Most of the constructs failed as the screw dislodging accompanied with cortex disruption

All of the specimens completed the entire cyclic loading test. Afterwards, the specimens were loaded until catastrophic failure. Based on the load-displacement curve, all the specimens exhibited changes in the curve slope prior to catastrophic failure, which indicated loss of fixation or plastic deformation of the construct. Followed with the plastic deformation, the maximum load detected by the testing machine was defined as the ultimate failure load, which was 1214 ± 127 N in the group A and 1109 ± 156 N in the group B. There were no significantly differences between the groups (*p* = 0.172). The most common pattern of failure was screw cut through the cancellous bone progressively, after that, the cortex bone was ruptured while maximum failure load was detected. (Fig. 4).

## Discussion

In this time zero study, the biomechanical properties of two different types of screw trajectories for the treatment of a lateral Hoffa fracture were compared. On the basis of the results, the crossed screw construct exhibited comparable biomechanical properties to those of the A-P screw construct. Therefore, the principle findings of the current study suggest that both screw trajectories will provide sufficient stability in terms of fracture fixation.

Several fixation methods, including screws and plates, have been utilized in the Hoffa fracture treatment. Sun et al. conducted a biomechanical analysis of four fixation constructs, and they concluded that a combination of plate and screw fixation could provide greater biomechanical stability than a screw fixation system alone [8]. In spite of its mechanical superiority, the popularity of plate fixation is still low given the requirement for extensive dissection and blood supply disturbance. Meanwhile, the ideal size and number of screws were discussed in a previous study [12]. A cadaveric study compared the biomechanical characteristics for three sizes of screws in a Hoffa fracture fixation, where the ultimate failure was significantly higher for 6.5-mm cancellous screws as compared to 3.5-mm cortical screws [21]. Furthermore, a biomechanical study showed that single 6.5 mm screws provide better fixation than either single or double 3.5 mm screws, yet a two screw construct is suggested to achieve two points of fixation in order to prevent fragment rotation [12]. Therefore, we utilized two 6.5 mm partially threaded screws in different trajectories in the current study.

The ideal screw trajectory has been discussed widely in previous studies for A-P screws, P-A screws, and crossed screws [19–21]. A biomechanical study conducted by Jarit et al. demonstrated that screws positioned in the P-A fashion exhibit significantly higher stability than A-P screws [11]. However, it is difficult to insert a P-A screw perpendicularly to a Hoffa fracture using a direct lateral approach. Therefore, an additional posterior approach is necessary [22], which inevitably increases the risk of common peroneal nerve injury or a compromise in blood supply [23]. In addition, fixation using a P-A screw necessitates advancing screws through the posterior condylar cartilage area, which will damage the weight-bearing articular surface [23]. Alternatively, Xu et al. treated eleven Hoffa fracture patients using crossed screws, and a comparison with traditional screws showed no significant differences in the function outcomes after 2 years of follow-up, which might indicate that crossed screws are as effective as traditional screws [19, 20].

The surgical approach depends on the pattern of the fracture or surgeon preference. When a Hoffa fracture occurs in association with a distal femoral fracture, both surgical approaches, including the direct lateral or lateral parapatellar approach, are commonly used. For fixation of a Hoffa fracture, an A-P screw is easier to apply via the parapatellar approach. However, an anatomic study showed that when the Hoffa fragment is less than 10.1% of the AP diameter of the lateral condyle, the parapatellar approach does not allow direct visualization of the fracture [23], which makes reduction difficult and may result in unsatisfactory results. In contrast, the direct lateral approach may provide better access to the Hoffa fracture. Shi et al. reported excellent results in 12 isolated lateral Hoffa fractures treated through the direct lateral approach [18]. In addition, a crossed screw could be inserted from the nonarticular area of the fractured condylar fragment in this approach. On the basis of our results, it was determined that a crossed screw may offer similar fixation strength as compared to a traditional A-P screw.

There are some limitations to this study. First, the fracture model was set in a synthetic bone rather than in a cadaveric femur, which might be better to simulate an in vivo situation. However, the uniform geometry of synthetic bone could minimize interspecimen variations in the physical properties of the bone. Also, the synthetic composite femur utilized in the current study was designed in accordance with the mechanical properties of a healthy male < 60 years of age [25]. Given that the current study was mainly aimed at high-energy trauma occurring in a younger population, synthetic bone could prevent the complications associated with inherent osteoporosis that might be a characteristic of cadaveric specimens. Second, not all of the force components were included in the biomechanical test. For example, bending and torsion tests were not conducted in the current study. Finally, P-A screws or plates were not included in the current study. As described previously, we focused on the crossed screw trajectory because it could be inserted using a direct lateral approach, and given that a comparison of A-P and P-A screws was made in a previous study, too many groups in the study might have led to confusing results.

## Conclusion

Based on our in vitro study, the crossed screws can provide comparable mechanical performance as traditional A-P screws in Hoffa fracture fixation. Considering the screws trajectories are commonly determined by the choice of surgical approach, the current study provides support from a biomechanical perspective for the application of crossed screws in direct lateral approach.

## Data Availability

All relevant data supporting the conclusions are included within the article and tables. The datasets used and/or analysed during the current study available from the corresponding author on reasonable request.

## Abbreviations

A-P screw: Anteroposterior direction screw
P-A screw: Posteroanterior direction screw
DLA: Direct lateral approach
PPA: Parapatellar approach
